# Pipeline for precise insoluble matrisome coverage in tissue extracellular matrices

**DOI:** 10.3389/fbioe.2023.1135936

**Published:** 2023-05-22

**Authors:** Wei Chen, Wen Zhang, Ning Zhang, Shuyan Chen, Tao Huang, Hong You

**Affiliations:** ^1^ Beijing Clinical Research Institute, Beijing, China; ^2^ Experimental and Translational Research Center, Beijing Friendship Hospital, Capital Medical University, Beijing, China; ^3^ Liver Research Center, Beijing Friendship Hospital, Capital Medical University, Beijing, China; ^4^ Beijing Key Laboratory of Translational Medicine in Liver Cirrhosis, National Clinical Research Center of Digestive Diseases, Beijing, China

**Keywords:** decellularization, matrisome, sodium dodecyl sulfate, LC-MS/MS, proteomic

## Abstract

The extracellular matrix (ECM) is assembled by hundreds of proteins orchestrating tissue patterning and surrounding cell fates via the mechanical–biochemical feedback loop. Aberrant ECM protein production or assembly usually creates pathological niches eliciting lesions that mainly involve fibrogenesis and carcinogenesis. Yet, our current knowledge about the pathophysiological ECM compositions and alterations in healthy or diseased tissues is limited since the methodology for precise insoluble matrisome coverage in the ECM is a “bottleneck.” Our current study proposes an enhanced sodium dodecyl sulfonate (E-SDS) workflow for thorough tissue decellularization and an intact pipeline for the accurate identification and quantification of highly insoluble ECM matrisome proteins. We tested this pipeline in nine mouse organs and highlighted the full landscape of insoluble matrisome proteins in the decellularized ECM (dECM) scaffolds. Typical experimental validations and mass spectrometry (MS) analysis confirmed very little contamination of cellular debris remaining in the dECM scaffolds. Our current study will provide a low-cost, simple, reliable, and effective pipeline for tissue insoluble matrisome analysis in the quest to comprehend ECM discovery proteomic studies.

## Introduction

The extracellular matrix (ECM) is a three-dimensional (3D) non-cellular network composed of macromolecules and sequestered growth factors that is present in almost all tissues. Bioactive ECM members and mechanical force offer multiple inputs into local intracellular biochemical signaling via cell surface adhesion receptors, mainly integrins, orchestrating tissue patterning and surrounding cell fates such as shape, proliferation, differentiation, polarity, motility, and survival (Hynes and Naba, 2012). Upon chronic or severe insults, tissue stiffening and mechanical stress coevolve with excessive synthesis and/or cross-linking of ECM proteins, creating pathological ECM niches that initiate and exacerbate various diseases, primarily fibrogenesis and carcinogenesis, via an aberrant mechanical–biochemical feedback loop between the ECM and its circumjacent cells (Chen et al., 2019; Henke et al., 2019; Pehrsson et al., 2021; Tzanakakis and Nikitovic, 2022). To this end, the ECM discovery proteomic analysis is currently encouraged to uncover differences between case–control tissues with the aim to better explore pathological mechanisms, prognostic biomarkers, or promising therapeutic targets (Taha and Naba, 2019).

Nowadays, putative proteins composing the ECM scaffold termed “matrisome” have been defined using *in silico* and proteomic approaches and have broadened to comprise not only collagens, glycoproteins, and proteoglycans but also ECM-affiliated proteins, ECM regulators, and secreted factors latent in ECM (Naba et al., 2016; Shao et al., 2020). Mass spectrometry (MS) acquisition is a typical approach for in-depth proteome coverage of tissues, but it usually achieves low efficiency for matrisome quantification from protein extraction due to ∼80% of the core ECM remaining in the sediment that is always discarded (Hill et al., 2015). Most of the core matrisome proteins possess large molecular weights and are covalently cross-linked, thus rendering them mostly insoluble and resistant to extraction even with the strongest detergents (Barrett et al., 2017; Krasny and Huang, 2021). Given this, ECM protein enrichment protocols prior to MS analysis have been proposed, typically consisting of a physical, chemical, or enzymatic treatment-mediated decellularization step followed by a Lys-C and/or trypsin-catalytic proteolytic digestion process (McCabe et al., 2021; Solarte David et al., 2022).

Although these methodologies have been proposed in the application of tissue decellularization, assessments by using traditional histological staining or genomic DNA quantification highlight that sodium dodecyl sulfonate (SDS) treatment in particular yields the highest ECM purity with the lowest contamination of cellular debris and nuclear remnants (Krasny and Huang, 2021). But when MS profiling is applied to matrisome quantification, it is surprising to find ∼50% of the total protein abundance in SDS decellularized ECM (dECM) scaffolds is actually non-matrisome (Krasny et al., 2016), suggesting that the current SDS decellularization is not sufficient to obtain pure ECM enrichment for precise proteome coverage. Because the tissue ECM scaffold is assembled by matrisome members with different gradients of solubility, it is hard to precisely characterize all fractions in the extracellular space. Therefore, a thorough removal of the cellular components embedded in the ECM prior to proteomic profiling is an alternative yet urgent requirement to fully characterize the highly insoluble matrisome atlas. To achieve this goal, our present study developed a pipeline for complete decellularization, proteolytic digestion, and MS quantification based on the SDS decellularization method proposed by Baiocchini et al., (2016). This pipeline was tested in nine organs or small pieces of tissues from mice, namely, the adipose, brain, duodenum, heart, kidney, liver, lung, spleen, and stomach to verify its efficiency.

## Methods and materials

### Reagents

The primary reagents and materials used in this study are listed as follows: acetonitrile (34851, Sigma-Aldrich); Alexa Fluor 488–labeled donkey anti-rabbit secondary antibody (A21206, ThermoFisher Scientific); Alexa Fluor 488–labeled donkey anti-mouse secondary antibody (A21202, ThermoFisher Scientific); ammonium bicarbonate (A643-500, Fisher Scientific); anti-collagen I primary antibody (1:100, 72026, CST); anti-collagen III primary antibody (1:100, ab7778, Abcam); anti-collagen VI primary antibody (1:200, ab182744, Abcam); anti-elastin primary antibody (1:50, MAB2503, Millipore); anti-GAPDH primary antibody (1:800, ab8245, Abcam); anti-LOXL1 primary antibody (1:50, sc-166632, Santa Cruz); anti-MAGP1 primary antibody (1:50, sc-166075, Santa Cruz); carbon tetrachloride (CCl_4_, 10006418, Sinopharm Chemical Reagent Co., Ltd.); dithiothreitol (DTT, Prod 20290, ThermoFisher Scientific); donkey serum (SL050, Solarbio); the elastin van Gieson (EVG) staining kit (EVG-010, BASO); fast green dye (IF0020, Solarbio); formic acid (27001, Sigma-Aldrich); glutaraldehyde (P1126, Solarbio); HPLC-grade water (HP9021LT, Fisher Scientific); iodoacetamide (ICN10035105, Fisher Scientific); Lys-C (125-05061, Wako); NaCl (S9888-500G, Sigma-Aldrich); optimal cutting temperature (OCT) compound (SAKURA, Japan); PBS buffer (D8537, Sigma-Aldrich); the Pierce BCA protein assay kit (23227, ThermoFisher Scientific); PNGase F, peptide-N-glycosidase F (P0704L, BioLabs); protease inhibitor cocktail (20124ES03, Yeasen); the Residual SDS Assay Kit (C500055, YaJi Biological); SDS (BP166-500, Fisher Scientific); Sirius Red dye (S8060, Solarbio); the Tissue Genomic DNA Extraction Kit (DP304-02, TIANGEN); trifluoroacetic acid (A116-10X1AMP, Fisher Scientific); Tris-base (T8060, Solarbio); Triton X-100 (T8200, Solarbio); Trypsin (V511A, Promega); Tween 20 (T8220, Solarbio); and urea (U6504, Sigma-Aldrich). All solutions were prepared in HPLC-grade water or in dedicated buffers.

### Mouse tissue collection

Wild-type C57BL/6J mice (10 weeks, male, purchased from Beijing HFK Bioscience Co., Ltd.) were euthanized by neck dislocation under anesthesia. The visceral adipose, brain, duodenum, heart, kidney, liver, lung, spleen, and stomach were surgically dissected and stored at −80°C until use. Small pieces of tissues were cut, weighted, placed into precooled tubes, and frozen in liquid nitrogen, and then stored at −80°C until use. The liver tissues from the control, liver fibrosis, and fibrosis resolution mouse models established in our previous study (Chen et al., 2019) were used for decellularization. Briefly, collagen α1(I)-GFP mice (male, 8 weeks old) were intraperitoneally injected with 12.5% carbon tetrachloride (CCl_4_) in olive oil (1/7, v/v) at a dose of 0.01 mL/g body weight twice a week for 12 weeks (defined as “liver fibrosis”). Liver fibrosis mice subsequently underwent spontaneous recovery for another 12 weeks (defined as “fibrosis resolution”) after the cessation of CCl_4_ intoxication. Control mice received an equal volume of olive oil for 12 weeks (defined as “control”). All mouse studies were approved by the Ethics Committee of Beijing Friendship Hospital, Capital Medical University, and carried out in accordance with the ARRIVE guidelines.

### Decellularization

**FIGURE 1:**
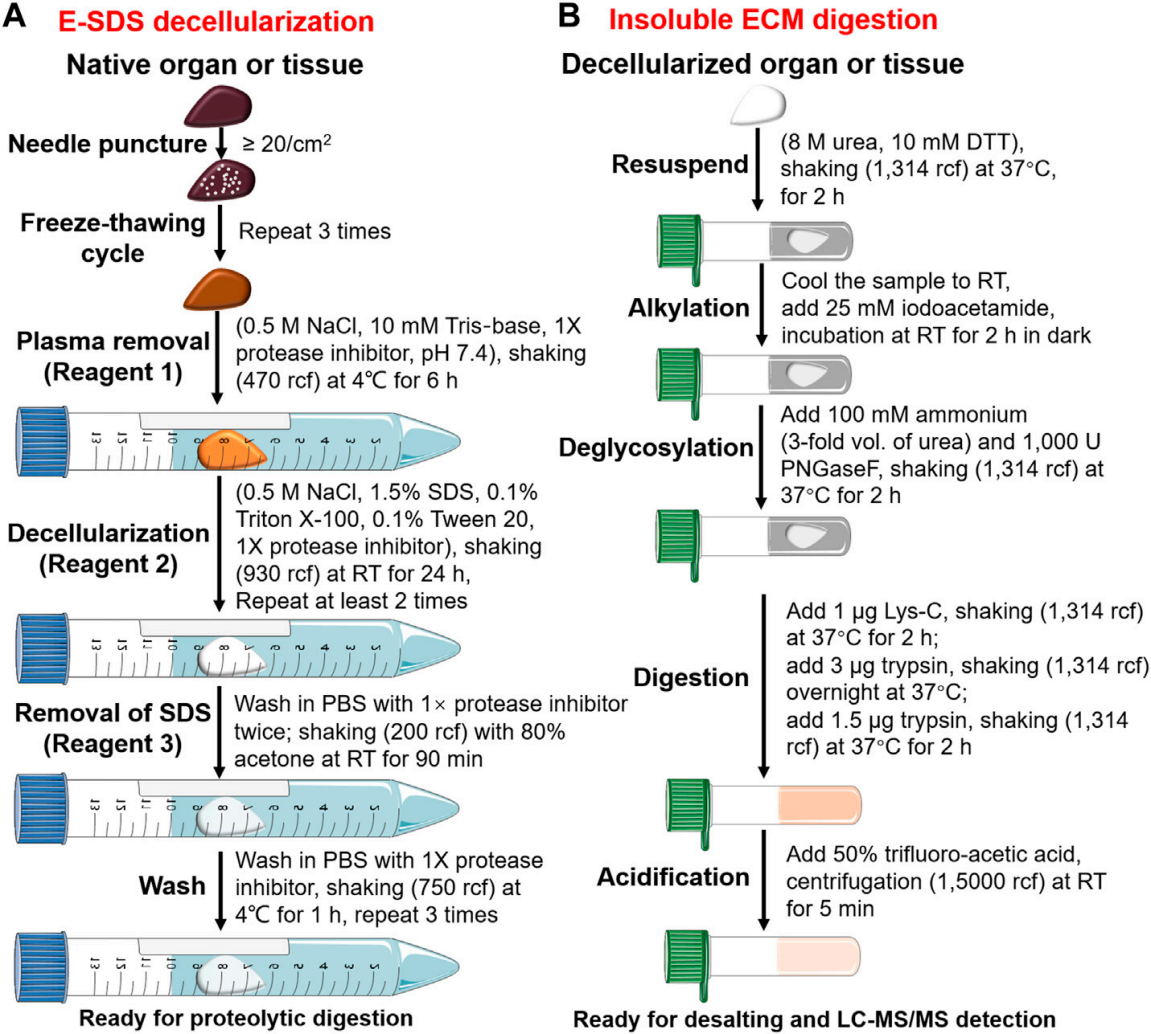
Pipeline of E-SDS decellularization and proteolytic digestion. Schematic workflow depicting the main steps for **(A)** ECM scaffold purification using E-SDS decellularization and **(B)** subsequent enzyme digestion of insoluble ECM proteins.

SDS decellularization workflow was modified based on Baiocchini et al. (2016) (Supplementary Material). We defined it as the enhanced SDS (E-SDS) method, which is summarized in Figure 1A and listed below.

• Needle puncture: small pieces of tissues (∼1 cm^3^) or whole organs were punctured (∼20 times/cm^2^) using the needle of 1 mL injector (0.5 mm × 16 mm, BD, United States). *Note: a needle puncture should be executed on a precooled metal plate or culture dish placed on ice and completed as quickly as possible.*
• Freeze–thawing cycle: tissues or whole organs that underwent needle puncture were immediately immersed in liquid nitrogen for 1 min and then kept at room temperature (RT) until completely thawed. Three cycles were repeated. *Note: freezing temperatures can be as low as −196°C and thawing temperatures as high as 37°C*.
• Plasma removal: plasma removal was carried out using Reagent 1 solution (0.5 M NaCl, 10 mM Tris-base, 1× protease inhibitor in HPLC-grade water, pH = 7.4) upon agitation using a rotary vibrator (470 relative centrifugal force [rcf]) at 4°C for at least 6 h. Pellets were retained for subsequent decellularization after rapid centrifugation (4°C, 15,000 rcf, 1 min). *Note: larger Eppendorf tubes with more than two-thirds volume of Reagent 1 solution were used to ensure tissues or organs inside shook freely and adequately. Protease inhibitor is diluted freshly when it is used*.
• Decellularization: the resulting pellets were then decellularized with Reagent 2 solution (0.5 M NaCl, 1.5% SDS, 0.1% Triton X-100, 0.1% Tween 20 and 1× protease inhibitor in HPLC-grade water), shaking (930 rcf) at RT for 24 h. Samples were then centrifuged (4°C, 12,000 rcf, 1 min), and pellets were retained. This step was repeated at least twice until the samples became white, off-white, or transparent. *Note: pre-prepared Reagent 2 should be placed at RT and confirmed with no sediments before use. A protease inhibitor is diluted freshly when it is used. Also, an Eppendorf tube with more than two-thirds volume of Reagent 2 solution is used to ensure tissues or organs inside shake freely and adequately. The decellularization duration depends on tissue nature*.
• Removal of residual SDS: the dECM scaffold was washed twice in PBS with 1× protease inhibitor and then incubated with 5 mL of Reagent 3 solution (80% acetone in HPLC-grade water) upon agitation (200 rcf) at RT for 90 min.
• The wash: the dECM scaffold was washed in PBS with 1× protease inhibitor under automatic shaking (750 rcf) at RT for 1 h and then centrifuged (4°C, 12,000 rcf, 1 min), and pellets were retained. The wash process was repeated thrice. *Note: protease inhibitors are diluted freshly when they are used*.

### Proteolytic digestion

Proteolytic digestion was performed according to previous reports (Naba et al., 2015; Dussoyer et al., 2022). A brief workflow is shown in Figure 1B and described as follows.

• Reduction: decellularized dECMs (∼5–10 mg dry weight) were resuspended and reduced in an 8 M urea solution (50 μL) with 10 mM DTT upon continuous agitation (1,314 rcf) at 37°C for 2 h.
• Alkylation: pre-prepared iodoacetamide solution (500 mM) was diluted to a final concentration of 25 mM in urea solution (pre-cooled to RT) and incubated in the dark at RT for 30 min.
• Deglycosylation: the abovementioned urea solution was diluted to 2 M by adding 150 μL of 100 mM ammonium bicarbonate (pH = 8.0). ECM proteins were deglycosylated using 1,000 U PNGase F at 37°C for 2 h with continuous agitation (1,314 rcf).
• Digestion: deglycosylated ECM proteins were digested into peptides by treating with 1 μg of Lys-C for 2 h, followed by 3 μg of trypsin overnight. The next day, trypsin (1.5 μg) was added and incubated for another 2 h. All enzyme digestion steps underwent continuous shaking (9,39 rcf) at 37°C.
• Acidification: digestion was terminated by freshly prepared 50% trifluoroacetic acid (TFA) at pH ≤ 2.0. Acidified samples were centrifuged (15,000 rcf) at RT for 5 min. The supernatant of the peptide was stored at −80°C or used directly.
• Desalting: Sep-Pak C18 column (WAT054955, Waters) was activated by 100% acetonitrile, equilibrated by 0.1% formic acid, and then loaded with the peptide solution. The Sep-Pak C18 column was washed using 0.1% formic acid to remove impurities and eluted using 70% acetonitrile. The flow-through was freeze-dried in a vacuum freeze dryer, dissolved in 2% acetonitrile/0.1% formic acid, and quantified by using the Pierce BCA Protein Assay Kit per the manufacturer’s protocol.

### Validation of decellularization efficiency

• Gross inspection: morphology and color of samples were recorded and photographed every 6 h during the decellularization process.
• Fast Green and Sirius Red (FG and SR) staining: paraffin-embedded native or decellularized tissue sections (7 μm) were incubated in 0.04% Fast Green for 15 min. After washing with PBS, the sections were treated with 0.1% Fast Green and 0.04% Sirius Red in saturated picric acid for 30 min. Images were photographed after mounting coverslips. The non-collagenous part appears green, whereas the collagenous fiber appears red.
• Elastic fiber staining: elastic fiber in paraffin-embedded sections (7 μm) was visualized using a commercial EVG staining kit according to the vendors' protocols.
• Genomic DNA quantification: as previously reported by Wang et al., (2015); Ohata and Ott, (2020); Mora-Navarro et al., (2021), we used absorbance spectroscopy by monitoring the representative DNA release profile from the tissue into the decellularization effluent at 260 nm in real time. Briefly, decellularized solutions from all the tissues (four replicates for each tissue) were retained at the point of 24 h (first round of SDS decellularization) and 48 h (second round of SDS decellularization) for DNA quantification by BioSpec-nano (Shimadzu). DNA in native tissues or dECM scaffolds was extracted using a commercial Tissue Genomic DNA Extraction Kit per the manufacturer’s protocols and quantified using BioSpec-nano (Shimadzu). The absolute DNA content was calculated by multiplying the DNA concentration and volume and then dividing by tissue weight.
• Residual SDS test: residual SDS in dECM scaffold was measured using a commercial Residual SDS Assay Kit according to the vendor’s instructions. The absolute SDS content was calculated by multiplying SDS concentration and volume and then dividing this by tissue weight.
• Immunofluorescence: the liver dECM scaffolds from our E-SDS or protocol (Baiocchini et al., 2016) were immediately embedded in OCT compound and cut into 7-μm-thick slices. Frozen sections were blocked with donkey serum for 1 h at RT, incubated overnight at 4°C with anti-collagen III, collagen VI, elastin, GAPDH, LOXL1, or MAGP1 primary antibodies at indicated dilutions, and then conjugated with Alexa Fluor 488-labeled donkey anti-rabbit or mouse secondary antibodies at a dilution of 1:500 for 1 h in the dark. After washing with PBS, the sections were mounted for microscopic analysis.

### Validation of collagenous fiber integrity

• 3D immunofluorescence: immunofluorescent staining of collagen I was performed in frozen 20-μm tissue sections. To specifically visualize collagen I, confocal z-stacks were captured at 0.3-μm increments between z-slices using a laser scanning confocal microscope (Olympus); z-slice contours were merged into a 3D contour surface by using the FV10-ASW 4.2 viewer software (Olympus) as previously described (Chen et al., 2019).
• Scanning electron microscopy (SEM): liver dECM scaffold based on E-SDS decellularization from control, liver fibrosis, and fibrosis resolution mouse models were fixed in 2% paraformaldehyde and 2.5% glutaraldehyde (4°C, overnight). The next day, fixed dECM scaffold was mildly washed at least twice with PBS, followed by dehydration using a series of ethanol solutions with increasing concentrations (70%, 80%, 90%, and 100%) for 10 min/each concentration at RT. The dehydrated dECM scaffold was then placed in acetone and dried using a critical point dryer (Leica EM CPD300) with CO_2_. Next, the samples were installed on an aluminum stub, paint-coated with Au/Pd using an ion sputtering apparatus (Hitachi), and mounted for imaging on a scanning electron microscope (Hitachi).

### Label-free liquid chromatography–tandem mass spectrometry (LC-MS/MS)

A nanoflow LC-MS/MS system was carried out using a quadrupole Orbitrap mass spectrometer (Orbitrap Eclipse, ThermoFisher Scientific) coupled online through an EASY-nLC 1,200 ultra-high pressure system (ThermoFisher Scientific) via a nano-electrospray ion source. Peptide samples (two replicates for each tissue) were loaded at 500 ng per injection on a 25 cm column (150-μm inner diameter, packed with ReproSil-Pur C18-AQ 1.9-µm silica beads; Beijing Qinglian Biotech Co., Ltd.), and were separated using a gradient from 6% to 12% solvent for 15 min, then 12%–30% solvent for 48 min, and stepped up to 40% solvent for 10 min, followed by a 10 min wash at 95% solvent at 300 nL/min (solvent: 80% acetonitrile and 0.1% formic acid). The total duration of the run was 85 min. An in-house-developed oven was used to keep the column temperature at 60°C. The MS instrument was operated in data-dependent acquisition mode, collecting MS spectra in the Orbitrap mass analyzer (resolution, 120,000; range, 350–2000 m/z) with an automatic gain control target of 4E^5^ and a maximum ion injection time of 50 ms. Following higher energy collisional dissociation with a normalized collision energy of 30%, MS/MS spectra were collected in the Orbitrap (15,000 resolution) with an AGC target of 5E^4^ and a maximum ion injection time of 22 ms.

### MS data preprocessing

The Proteome Discoverer suite (version 2.4, Thermo Fisher Scientific) was employed for raw data analysis. Tandem mass spectra were searched against the UniProtKB mouse proteome database, which contains both Swiss-Prot and TrEMBL mouse reference protein sequences (55,315 target sequences downloaded on 17 March 2022). The SEQUEST-HT search engine was used with the following parameters: fully tryptic specificity, maximum of two missed cleavages, minimum peptide length of six, fixed carbamidomethylation of cysteine residues (+57.02146 Da), variable modifications for oxidation of methionine residues (+15.99492 Da), precursor mass tolerance of 15 ppm and fragment mass tolerance of 0.02 Da for MS2 spectra collected in the Orbitrap. Peptide spectral matches and peptides with a false discovery rate (FDR) of less than 1% were filtered by using the percolator. After spectral assignment, the peptides were assembled into proteins and further filtered based on the combined probabilities of their constituent peptides to a final FDR of 1%. By default, the top matching protein or ‘master protein’ is the protein with the largest number of unique peptides and with the smallest value in percent peptide coverage. Only unique and razor (that is, parsimonious) peptides were considered for quantification. Protein expression matrices were deposited in the figshare database (DOI:10.6084/m9.figshare.22722343).

### Analysis of matrisome proteins

Proteins identified by LC-MS/MS were searched against mouse matrisome lists (http://matrisomeproject.mit.edu/) *in silico* defined (Shao et al., 2020) using gene IDs and followed by a final manual confirmation. The number of total proteins or matrisome proteins identified in each tissue by label-free LC-MS/MS was summarized. The matrisome number to total protein number ratio, matrisome abundance (sum of all the matrisome abundance) to total protein abundance (sum of all the protein abundance) ratio, number proportion of each matrisome category (ratio of the number of members in each matrisome category to the number of total matrisome members), and abundance proportion of each matrisome category (ratio of the total abundance of members in each matrisome category to total abundance of matrisome members) were calculated and compared. Venn analysis of matrisome proteins among nine mouse tissues was analyzed and visualized using the *UpSetR* (Conway et al., 2017) package. Internal interaction networks among common matrisome members in nine tissues were retrieved and visualized using the STRING database (https://cn.string-db.org/). The *ClusterProfiler* (Yu et al., 2012) package was used to identify the enriched Kyoto Encyclopedia of Genes and Genomes (KEGG) pathways. Heatmaps were drawn by using the *pheatmap* (https://CRAN.R-project.org/package=pheatmap) package. The MS/MS data from liver dECM scaffolds decellularized by E-SDS decellularization proposed by Baiocchini et al. (2016) were also compared.

### Statistical analysis

Data are shown as mean ± standard deviation or percentage. The significance of the differences between two groups was determined by the unpaired Student’s *t*-test, the Chi-square trend test, or Fisher’s exact test. A *p* < 0.05 was considered statistically significant. The R 3.6.3, GraphPad Prism 7 (GraphPad Software Inc.), and SangerBox tool (http://sangerbox.com/) were used for statistical and bioinformatics analyses.

## Results

### High decellularization efficiency and dECM purity of E-SDS pipeline

**FIGURE 2:**
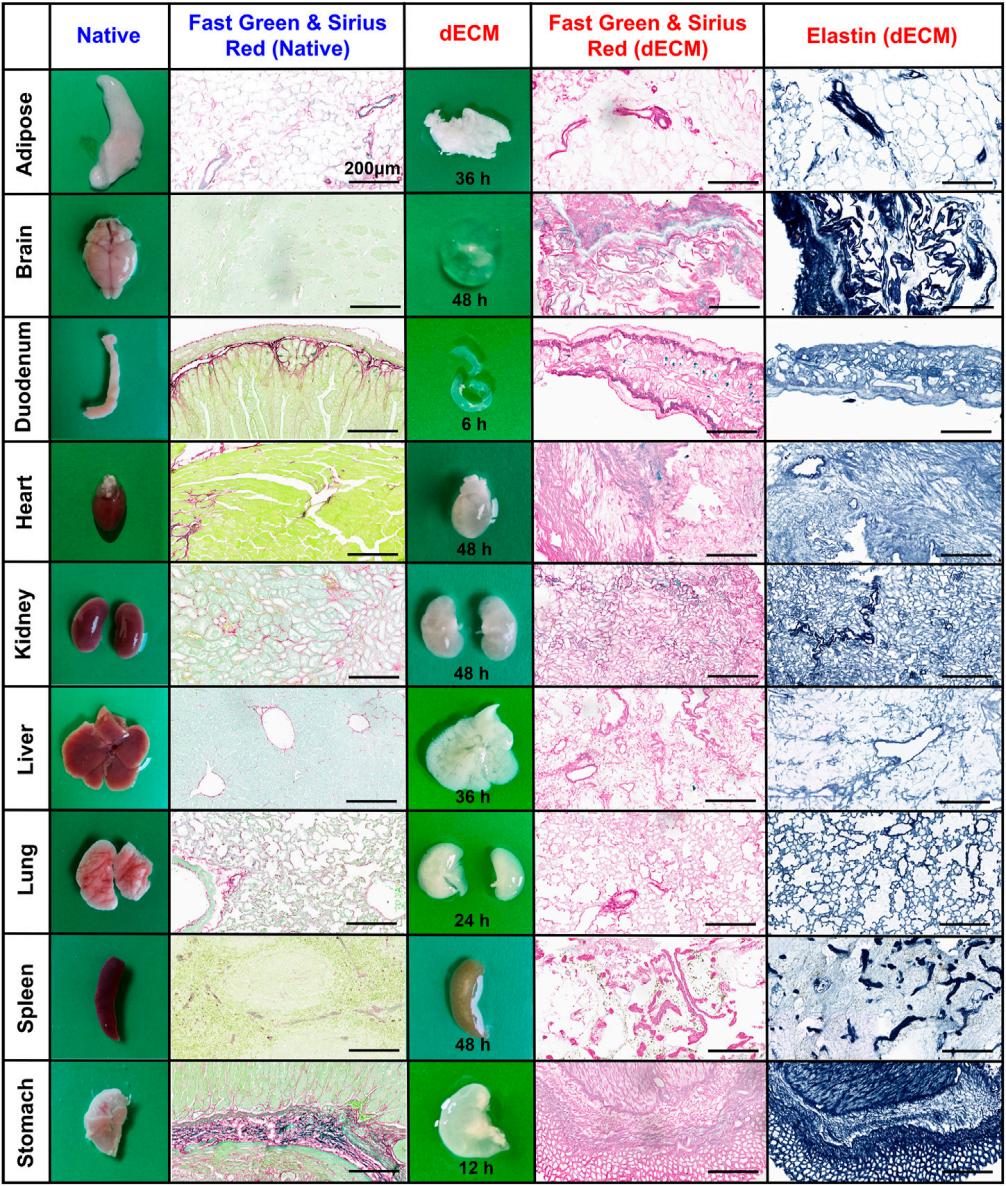
Histological assessment of dECM scaffolds purified by E-SDS decellularization. Representative images of morphology and color, and Fast Green and Sirius Red or elastin staining of resident cells (green/light blue), collagenous fiber (pink), and elastic fiber (blue) in native organs or their dECM scaffolds. The required time for complete decellularization was recorded. Scale bar: 200 μm.

All mouse organs undergoing E-SDS decellularization became white (adipose, liver, lung, and stomach), off-white (heart, kidney, and spleen), or transparent (brain and duodenum) within a relatively short time period ranging from 6 to 48 h; with the exception of the brain, dECM scaffolds structurally kept the morphology and innate microscopic structure of native organs (Figure 2). Apart from gross inspection, Fast Green and Sirius Red staining were used to evaluate the extent of cell remnant elimination. As shown in Figure 2, compared to the native organs, all the decellularized organs lost the characteristic green color of the resident cells, and the remaining dECM scaffolds appeared pink, indicative of a successful decellularization with substantial ECM enrichment and rare cell debris reservation. Furthermore, elastin staining also confirmed an enrichment of elastic fibers after decellularization.

**FIGURE 3:**
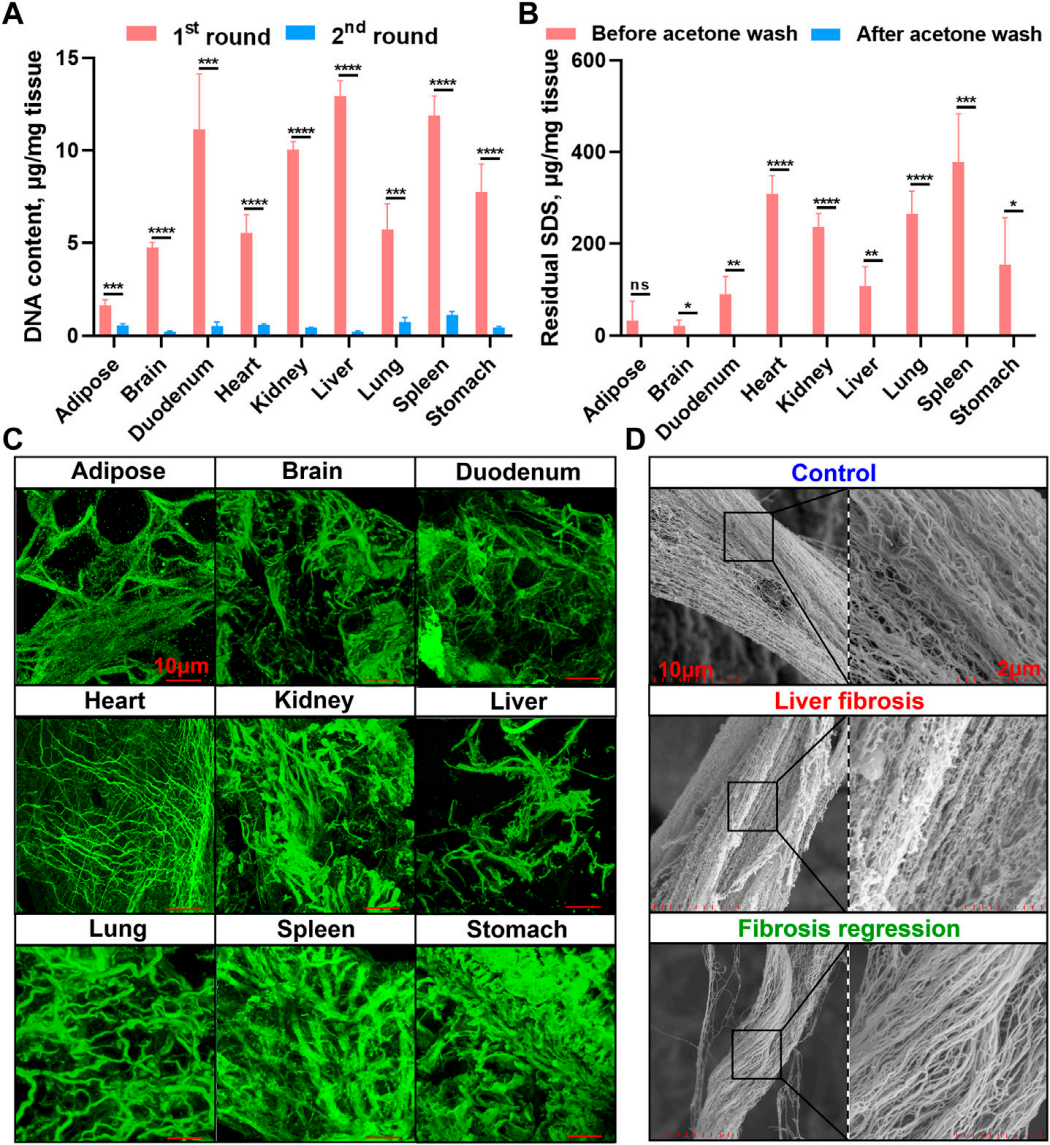
Genomic DNA quantification, residual SDS quantification, and collagen fiber visualization in dECM scaffolds. **(A)** Spectrophotometric quantification of DNA content in solutions from the first (24 h) and second (48 h) rounds of E-SDS decellularization (*n* = 4 for each tissue). The absolute DNA content was calculated by multiplying DNA concentration and volume and then dividing by tissue weight. **(B)** Quantification of residual SDS in dECM scaffolds before and after acetone precipitation. The absolute SDS content was calculated by multiplying SDS concentration and volume and then dividing by tissue weight. Significance of difference was determined using Student’s *t*-test. *p < 0.05, **p < 0.01, ***p < 0.001, ****p < 0.0001, ns represents no significance. **(C)** 3D immunofluorescent visualization of collagen I staining in dECM scaffolds by laser scanning confocal microscopy. Scale bar: 10 μm. **(D)** Ultrastructural view of collagen I in dECM scaffolds obtained from control (olive oil), liver fibrosis (CCl_4_ intoxication for 12 weeks), and fibrosis regressive (CCl_4_ cessation for 12 weeks) mouse livers using SEM. Scale bar: 2 μm.

We next measured the nuclear materials in both native tissues and dECM scaffolds. In the first round of decellularization solution, the DNA content was highly detectable, whereas it was dramatically reduced in the second round solution in all the tissues, indicating decellularization mainly occurred in the first round of decellularization (24 h) (Figure 3A). A commercial tissue genomic DNA extraction kit was also applied to enrich DNA from native tissues and dECM scaffolds, but it revealed a significantly lower enrichment capacity of genomic DNA than when using the E-SDS method (Supplementary Figure S1). Taken together, the E-SDS pipeline has the potential to be used in mouse tissue or organ decellularization, achieving a relatively high efficiency in dECM scaffold purification.

### Lower SDS residue and higher collagenous fiber integrity in dECM scaffolds using E-SDS decellularization

Afterward, residual SDS content in the dECM scaffold was measured. As shown in Figure 3B, the dECM scaffolds before acetone precipitation contained higher levels of residual SDS, while SDS content was almost undetectable in dECM scaffolds after acetone wash, indicating that acetone has a strong capacity for precipitating SDS and that this step is indispensable to sufficiently reduce the cytotoxicity of E-SDS method–derived dECM scaffolds. Despite studies showing that the SDS detergent always elicited an altered microstructure and diminished ECM integrity (Xu et al., 2014), upon FG and SR and elastic staining (Figure 2), large empty spaces once occupied by cells could be clearly noticed (except in the brain dECM scaffold); collagenous and elastic fibers with different diameters were observed in all dECM scaffolds. Furthermore, a 3D reconstruction (Figure 3C) of specific immunostaining of collagen I clearly revealed collagenous fibers in the E-SDS-treated organs. We further performed SEM using the mouse liver dECM scaffold as an example. Ultrastructural analysis showed that the alignment and orientation of collagenous fibers were well preserved in the dECM scaffolds from control, fibrotic, and fibrosis-regressive mouse livers (Figure 3D). Given this, our E-SDS decellularization method is likely to have the potential to aid the accurate characterization of the structural information of collagen fibers in healthy or diseased tissue ECM, which may be of great interest to improve histological diagnosis.

### Highly insoluble matrisomes in dECM scaffolds from mouse tissues unveiled

**FIGURE 4:**
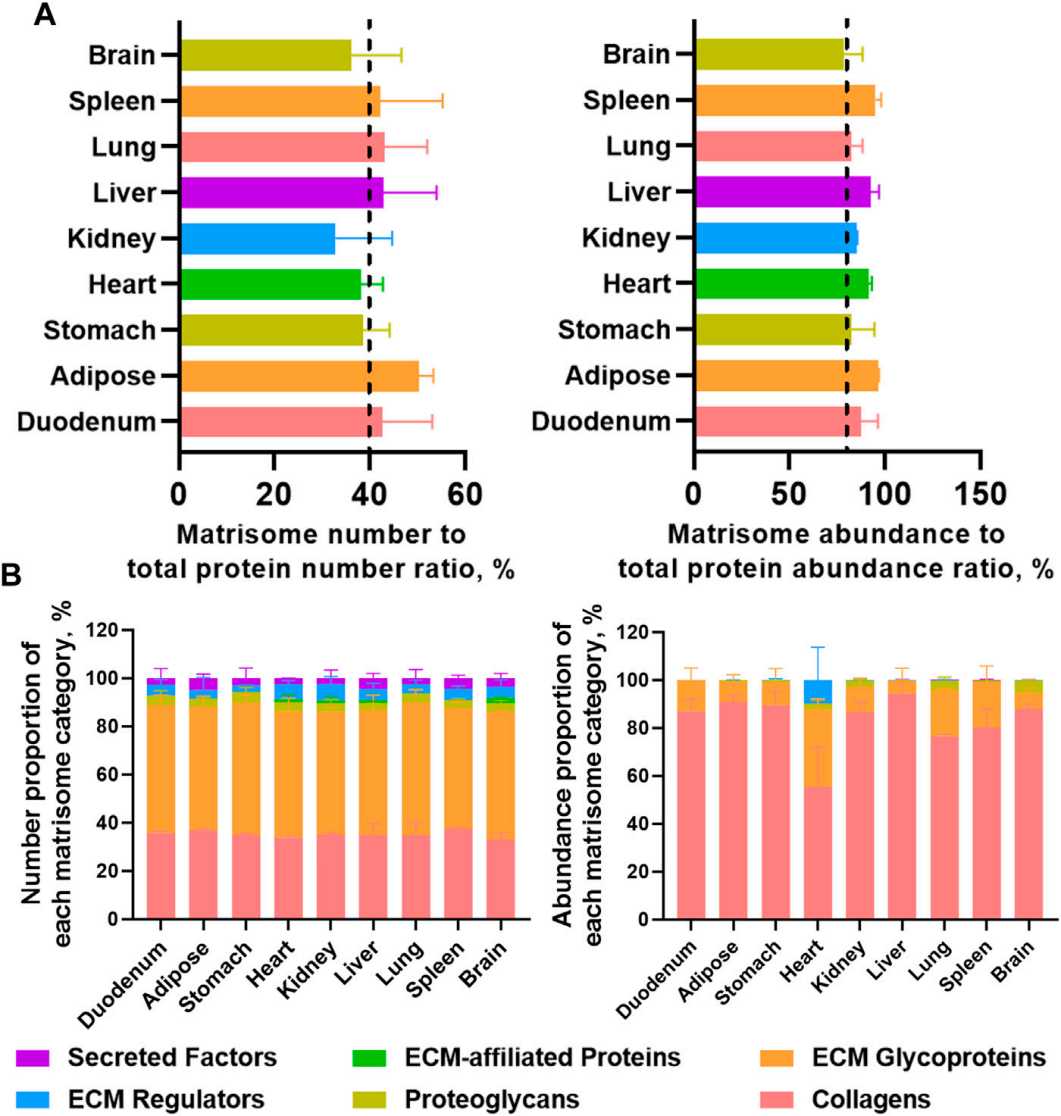
Highly insoluble matrisome proteins in dECM scaffolds from mouse tissues unveiled by label-free LC-MS/MS. **(A)** Matrisome number to total protein number ratio and matrisome abundance (sum of all the matrisome abundance) to total protein abundance (sum of all the protein abundance) ratio detected from each dECM scaffold by label-free LC-MS/MS. Different tissues are color coded. **(B)** Number proportion of each matrisome category (number of members in each matrisome category to number of total matrisome members ratio), and abundance proportion of each matrisome category (total abundance of members in each matrisome category to total abundance of matrisome members ratio). Matrisome categories are color coded. Data are presented as mean ± standard deviation (*n* = 2 for each tissue).

Label-free proteomics analysis allowed us to unveil highly insoluble matrisome proteins in the dECM scaffolds from mouse tissues (figshare, DOI: 10.6084/m9.figshare.22722343). The matrisome number to total protein number ratios in nine dECM scaffolds ranged from 32.8% to 50.3% (Figure 4A). However, the abundance of matrisome proteins accounted for the most, as the matrisome abundance to total protein abundance ratios ranged from 78.5% in the brain to as high as 96.2% in the adipose (Figure 4A), suggesting that the E-SDS method can retain more extracellular insoluble proteins, with the potential to be used in MS profiling of insoluble matrisome proteins in wide-ranging tissues. Specifically, although the number of proteoglycans accounted for the most across all mouse tissues (Figure 4B), comparable with a previous report (Krasny et al., 2016), collagens appeared as the most abundant insoluble matrisome proteins in all dECM scaffolds (Figure 4B).

### Comparison of insoluble matrisome proteins among different mouse tissues

**FIGURE 5:**
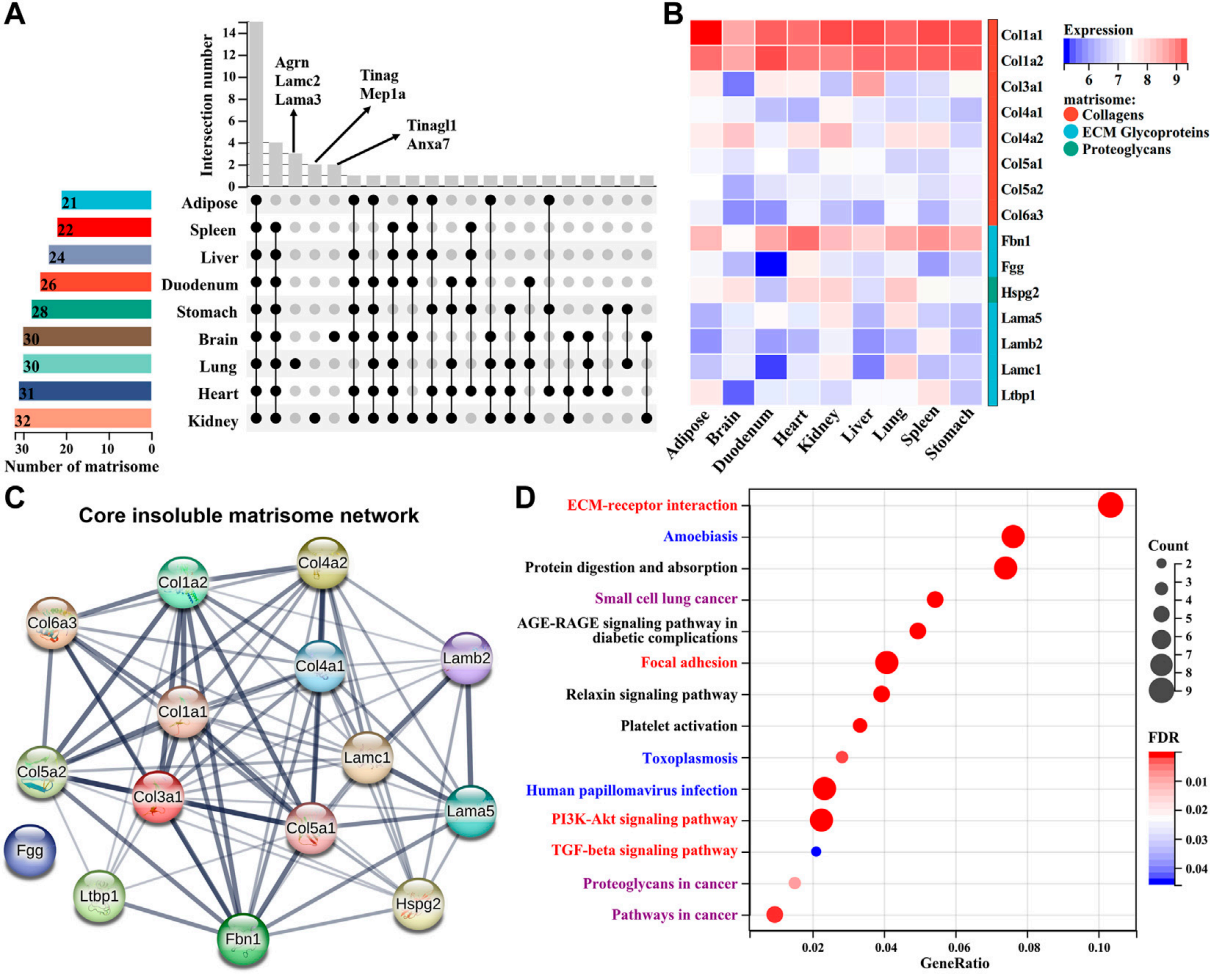
Comparison of insoluble matrisome proteins among nine mouse tissues. **(A)** UpSet diagram of insoluble matrisome proteins among nine dECM scaffolds. The vertical and horizontal axes represent the number and distribution of insoluble matrisome proteins in the indicated mouse dECM scaffold. **(B)** Heatmap of the abundance of commonly identified matrisome proteins in ECM scaffolds. Log2-transformed abundance values were scaled as a distribution with a mean = 0 and standard deviation = 1. The darker the blue, the lower the expression; the darker the red, the higher the expression. Matrisome categories are color coded. **(C)** The potential interaction network among the common insoluble matrisome proteins retrieved from the STRING database. The interactive relationship between two proteins is connected by an edge. **(D)** Significantly enriched KEGG pathways of the commonly identified matrisome proteins in nine ECM scaffolds. The size of the circle represents the number of matrisome proteins, and the color of the circle represents the adjusted *p*-value. An adjusted *p* < 0.05 was considered statistically significant. Fibrogenesis, immune, or proliferation-related KEGG pathways are highlighted as red, blue, or purple, respectively.

We next systematically compared the differences or similarities of insoluble matrisome proteins across nine mouse tissues. The common matrisome proteins in each tissue identified from two replicates were retained for subsequent analyses (Supplementary Figure S2). Most of the matrisome proteins were globally expressed across all the tissues, while a few of them exhibited a tissue specificity (Figure 5A). For instance, tubulointerstitial nephritis antigen (TINAG) and meprin A subunit alpha (MEP1A) were exclusively expressed in kidney dECM scaffold; laminin subunit gamma-2 (LAMC2), agrin (AGRN) and laminin, alpha 3 (LAMA3) were uniquely found in the lung dECM scaffold; tubulointerstitial nephritis antigen-like protein (TINAGL1) and annexin A7 (ANXA7) were only identified in the brain dECM scaffold. Except for tissue-specific matrisome members, a total of eight collagens, six ECM glycoproteins and one proteoglycan [heparan sulfate proteoglycan 2 (HSPG2)], were widely expressed across all tissues with variable abundance (Figure 5B). These common matrisome proteins highly interact with each other, probably constituting the core insoluble matrisome network in mouse tissues (Figure 5C). Functional enrichment analysis showed that these matrisome proteins were associated with fibrogenesis, immune, and proliferation–related KEGG pathways, covering the biological functions of the dECM scaffold (Figure 5D).

### Performance evaluation of E-SDS pipeline for precise insoluble matrisome coverage

**FIGURE 6:**
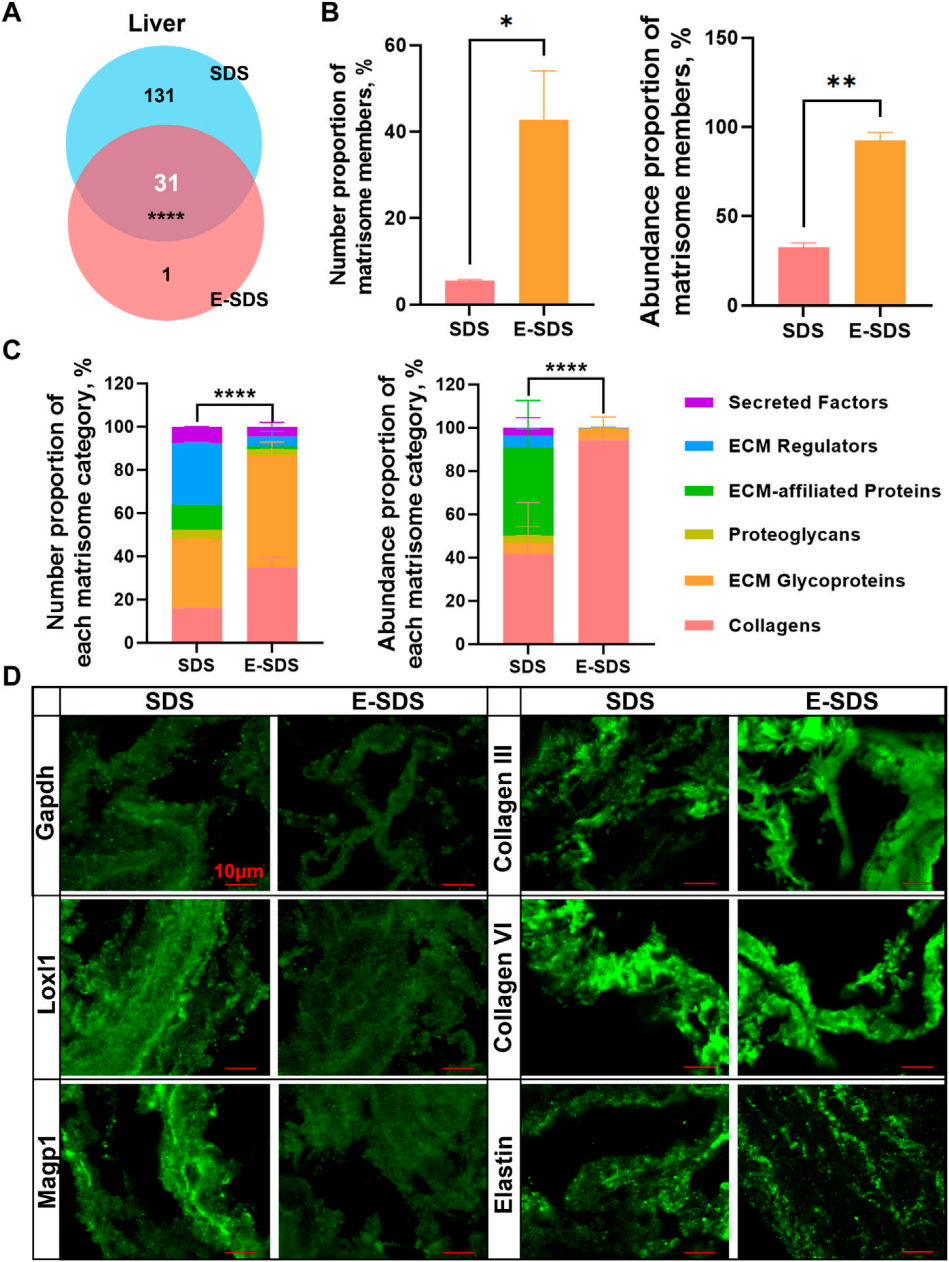
Performance evaluation of the E-SDS pipeline for precise insoluble matrisome coverage in mouse liver decellularization. **(A)** Venn diagram of the commonly or uniquely identified matrisome proteins in the liver dECM scaffold obtained by the E-SDS pipeline or Baiocchini’s SDS method. Statistical significance was determined using the Fisher’s exact test. ****p < 0.0001. **(B)** Comparison of the number or abundance proportion of matrisome members between liver dECM scaffolds derived by E-SDS pipeline or Baiocchini’s SDS method. Significance of difference was determined using Student’s *t*-test. *p < 0.05, **p < 0.01, *n* = 2 for each group. **(C)** Comparison of number or abundance proportion of each matrisome category between liver dECM scaffolds derived by E-SDS pipeline or Baiocchini’s SDS method. Matrisome categories are color coded. Data are presented as mean ± standard deviation (*n* = 2 for each tissue). **(D)** Comparison of collagen III, collagen VI, elastin, GAPDH, LOXL1, or MAGP1 expressions in liver dECM scaffolds derived by E-SDS pipeline or Baiocchini’s SDS method. Scale bar: 10 μm.

To assess the performance of the E-SDS method for precise insoluble matrisome coverage in tissue extracellular matrices, we compared dECM scaffolds using both E-SDS and Baiocchini’s SDS methods. Most of the identified matrisome proteins in the liver dECM scaffold using the E-SDS method were commonly identified by Baiocchini’s SDS method, and only nephronectin (NPNT) was exclusively detected by the E-SDS method (Figure 6A). The number proportion of matrisome members [42.8% (E-SDS) *versus* 5.5% (Baiocchini’s)] and abundance proportion of matrisome members [92.4% (E-SDS) *versus* 32.5% (Baiocchini’s)] were dramatically increased in the liver dECM scaffold using the E-SDS decellularization method (Figure 6B). Totally, the number or abundance of matrisome components from each category in the liver dECM scaffold using E-SDS and Baiocchini’s SDS methods was rather different (Figure 6C); in comparison, both the number and abundance of collagens and ECM glycoproteins accounted for most in E-SDS method–derived liver dECM scaffold (Figure 6C). Accordingly, it is likely that the E-SDS method could eliminate more ECM-affiliated proteins, ECM regulators, and secreted factors latent in the ECM scaffold than the Baiocchini’s SDS method. We further performed immunofluorescent staining to validate the results of LC-MS/MS proteomics. As shown in Figure 6D, it was nearly undetectable of intracellular glyceraldehyde-3-phosphate dehydrogenase (GAPDH, non-matrisome), indicating a rare cell debris reservation in liver dECM scaffold using both SDS methods; the secreted lysyl oxidase-like 1 (LOXL1, ECM regulators) and microfibrillar-associated protein 2 (MAGP1, ECM glycoproteins) were abundantly identified in the liver dECM scaffolds, derived from the Baiocchini’s SDS method but not the E-SDS method, which was comparable with the results of LC-MS/MS detection; the ECM structural proteins, such as collagen III, collagen VI, and elastin were highly enriched in liver dECM scaffolds using both SDS methods. Collectively, the E-SDS method coupled with proteomic profiling could identify and quantify the highly insoluble matrisome proteins in tissue extracellular matrices.

## Discussion

Tissue decellularization is a very promising approach that offers biological templates for organ engineering and transplantation. By now, a lot of methodologies have been developed, comprising physical, chemical, or biological treatments and their combinations (Solarte David et al., 2022). By contrast, chemical treatment, particularly the ionic detergent SDS treatment, is demonstrated as a very effective reagent applied for tissue decellularization (Luo et al., 2019; Krasny and Huang, 2021; Dussoyer et al., 2022). Considering SDS will disrupt the mechanical integrity and microstructure of the dECM scaffold, adversely affecting cytocompatibility and the ability to facilitate functional tissue replacement (White et al., 2017); multiple studies have endeavored to modify the current SDS workflow to make it applicable in cell seeding (He et al., 2017; Mallis et al., 2018; Asgari et al., 2021; Al-Hejailan et al., 2022). However, the balance between the removal of non-ECM materials and preservation of ECM nature still challenges the advancement of the SDS-mediated decellularization approach. Alternatively, uncovering the truly altered matrisome members in a pure-enough dECM scaffold from diseased tissues will contribute to more important discoveries in pathogenesis and diagnostic markers (Taha and Naba, 2019). Our current study therefore modified the Baiocchini’s SDS decellularization method with the aim to extremely eliminate all the soluble components as much as possible yet retain the highly pure insoluble ECM members for global insoluble matrisome characterization.

SDS with a concentration ≤1% was frequently applied in tissue decellularization, while subsequent global proteomic profiling confirmed vast contamination with non-matrisome proteins (Johnson et al., 2016; Krasny et al., 2016; Garcia-Puig et al., 2019). To some extent, the remnants in the dECM scaffold are likely to be attributable to insufficient decellularization. Recent studies have shown that cell removal intensity was somewhat SDS concentration dependent (White et al., 2017; Luo et al., 2019). We have raised the concentration of SDS to 1.5% in the E-SDS protocol with the expectation that a higher concentration of SDS might achieve more sufficient cell removal. In addition, widely used non-ionic detergents such as 0.1% Triton X and Tween 20 that have complementary roles in cell disruption and protein extraction with SDS were also added. Tween 20 is a non-ionic solubilizing agent of membrane proteins (Dresser et al., 2022), and Triton X-100 usually breaks cells by disrupting DNA–protein, lipid–lipid, and lipid–protein interactions, whereas SDS denatures proteins and solubilizes cellular and nucleic membranes while disrupting protein–protein interactions (Moffat et al., 2022). A combination of SDS with other non-ionic detergents has also been confirmed to more effectively remove cytoplasmic cytoskeletal proteins and vimentin than does SDS alone (Liu et al., 2018), highlighting the enhanced acellular ability of the mixture of non-ionic and ionic detergents. In addition, physical treatments such as needle puncture and freeze–thawing cycles were added at the beginning of the detergent treatment in order to promote the detergent solution's penetration into tissues to the greatest extent possible, thus enabling enough pore formation in the tissues of choice and ensuring sufficient contact with the decellularization reagent.

Previously reported SDS approaches could be successfully applied in tissue regeneration, illustrating that part of the ECM nature was reserved (He et al., 2017; Mallis et al., 2018; Asgari et al., 2021; Al-Hejailan et al., 2022). The E-SDS method-derived dECM scaffold also kept the architecture and nature of native organic ECMs. With the exception of the brain ECM, the morphology and innate microscopic structures of the other decellularized organs were consistent with their native organs; also, fibrillar meshwork with large empty spaces was clearly seen in the remaining dECM scaffolds under histological assessment. The possible reason why brain ECM structural integrity could not be kept after decellularization is that hyaluronan accounts for most in the neural interstitial matrix, and fewer fibrillar components after thorough decellularization are not enough to support the microstructure of the decellularized brain (Kim et al., 2018). Our in-depth proteomic analysis also supports a broad lacking of fibrillar collagenous contents in the brain (figshare, DOI: 10.6084/m9.figshare.22722343). Previous studies have shown that high concentrations of SDS would induce a reduction of elastin globules and other attachments (Narciso et al., 2022). In the E-SDS decellularization pipeline, although the concentration of SDS was raised 1.5 times, elastin staining and immunostaining of elastin still showed a preservation of insoluble elastin in the resulted dECM scaffolds. Also, it is well known that the residual amount of SDS left in the dECM scaffolds will lead to undesirable cytotoxicity toward implanted cells. In the E-SDS method, acetone is sufficient to remove residual SDS in dECM scaffolds. To sum up, E-SDS decellularization largely protects the microstructures of dECM scaffolds that may have limited cytotoxicity for tissue regeneration.

Enriched ECM pellets obtained by previously reported SDS methods were stated to have fewer intracellular materials assessed by traditional experiments such as histological staining and genomic DNA measurement. However, a lot of non-matrisome proteins were still detected in high abundance upon further in-depth proteomic profiling analysis (Johnson et al., 2016; Krasny et al., 2016; Garcia-Puig et al., 2019). We, therefore, analyzed the purity of ECM scaffolds obtained based on E-SDS decellularization by LC-MS/MS analysis. Although the number of identified matrisome proteins by MS analysis based on E-SDS decellularization was comparable with that identified by using previous SDS methods (Krasny et al., 2016; Garcia-Puig et al., 2019), the abundance proportion of matrisome proteins was dramatically increased. MS detection highlighted the similarity and differences of insoluble ECM components across nine mouse tissues, which included subclasses of ECM components spanning over >5 orders of magnitude from highly abundant collagens to lowly abundant ECM glycoproteins based on E-SDS decellularization. The common matrisome proteins in the dECM scaffolds were functionally enriched in the basic biological functions of ECM scaffolds covering fibrogenesis, immune, and proliferation–related signals, indicating that the E-SDS method to some extent reserved the basic matrisome components in the ECM. Our MS analysis also found that collagen type I alpha-1(I) and alpha-2(I) chains were the highest signals, and fibrillin-1 was also widely and abundantly expressed in almost all the tissue dECM scaffolds, in line with a recent report (Kawecki et al., 2022). More importantly, the number and abundance proportions of matrisome proteins were dramatically increased in dECM scaffolds using the E-SDS pipeline over Baiocchini’s SDS method. Collectively, MS analysis confirmed a higher purity and reservation of insoluble matrisome proteins when the E-SDS method was applied to decellularization.

In addition, the E-SDS method has some other advantages. First, the decellularization pipeline prior to MS analysis is low cost and easy to operate. The whole decellularization procedure is independent of *in situ* perfusion, and the main chemicals in the decellularization solution include the widely used ionic detergent SDS and the non-ionic detergents Triton X-100 and Tween 20, therefore it is an economical and feasible procedure requiring less technical expertise. The second merit of the E-SDS method is time saving. The Baiocchini’s SDS method usually takes at least 48 h for whole decellularization or longer in large and thick-walled tissue blocks (Hill et al., 2015; Baiocchini et al., 2016; Krasny et al., 2016; Carpino et al., 2019; Wishart et al., 2020). But the E-SDS method largely shortened the duration of complete decellularization, ranging from 6 to 48 h, dependent on the organ type. Moreover, decellularization reagents are always optimized in order to fit tissues with different volumes, shapes, and thicknesses and matrix density considerations (White et al., 2017). In our current study, we tested the E-SDS pipeline in nine mouse organs and small pieces of tissues, all of which demonstrated a higher purification of ECM enrichment, indicating that the E-SDS method has wide applicability in multiple tissues.

In conclusion, lowly abundant matrisome proteins were usually undetectable unless undergoing an ECM-enriched decellularization process; nevertheless, insufficient decellularization always induces nuclear and cytoplasmic waste in the remaining matrix, greatly decreasing the purity of matrisome components in the subsequent MS analysis (Mendibil et al., 2020; Solarte David et al., 2022). Our current study proposes an E-SDS decellularization workflow. It is low cost, is easy to operate, and has wide applicability in multiple tissues. Most importantly, the E-SDS pipeline has a more improved efficiency in the elimination of ECM-affiliated proteins, ECM regulators, and secreted factors latent in the ECM scaffold than the Baiocchini’s SDS method, which may be more applicable for precise coverage of highly insoluble matrisome members. We expect that the E-SDS pipeline will aid researchers in the field of ECM discovery and proteomic study.

## Data Availability

The data sets presented in this study can be found in online repositories. The names of the repository/repositories and accession number(s) can be found at https://figshare.com/articles/dataset/matrisome_expression_matrix/22722343, DOI: 10.6084/m9.figshare.22722343.

## Abbreviations

CCl_4_, carbon tetrachloride; dECM, decellularized ECM; DTT, dithiothreitol; ECM, extracellular matrix; E-SDS, enhanced sodium dodecyl sulfonate; EVG, elastin van Gieson; FDR, false discovery rate; FG and SR, Fast Green and Sirius Red; KEGG, Kyoto Encyclopedia of Genes and Genomes; LC-MS/MS, liquid chromatography–tandem mass spectrometry; MS, mass spectrometry; OCT, optimal cutting temperature; rcf, relative centrifugal force; RT, room temperature; SDS, sodium dodecyl sulfonate; SEM, scanning electron microscopy; TFA, trifluoroacetic acid; 3D, three dimensional.
