# Supplementary material for: Pipeline for precise insoluble matrisome coverage in tissue extracellular matrices
Source: Front Bioeng Biotechnol. 2023 May 22;11:1135936. doi: 10.3389/fbioe.2023.1135936 (PMC10239929; doi:10.3389/fbioe.2023.1135936)
Supplement: Supplementary file 1 [file DataSheet1.docx]

**Supplementary document:**

**Pipeline for Precise** **Proteome Coverage of Insoluble Matrisome in Tissue Extracellular** **Matrices**

Wei Chen^1,2,#^, Wen Zhang^3,4,#^, Ning Zhang^3,4^, Shuyan Chen^3,4^, Tao Huang^1,2^, Hong You^3,4^*

**TABLE OF CONTENTS**

1. **SUPPLEMENTARY EXPERIMENTAL PROCEDURES**
2. **SUPPLEMENTARY FIGURES**

**SUPPLEMENTARY EXPERIMENTAL PROCEDURES**

**SDS decellularization protocol reported by Baiocchini et al.**

**Step 1:** Removal of plasma proteins from tissues is performed by overnight shaking (600 rpm) in Reagent 1 (0.5 M NaCl, 10 mM Tris Base, pH 7.5, 1X protease inhibitor) at 4°C, followed by centrifuging at 13,000 rpm for 1 minute.

**Step 2:** Pellets are washed twice with Reagent 2 (1% sodium dodecyl sulfonate [SDS] in PBS, 1X protease inhibitor) and incubated overnight in Reagent 2 at room temperature (shaking at 800 rpm). After 24 hours, the supernatants are removed and fresh Reagent 1 is added to the tissues. This process is repeated until the tissues are completely decellularized.

**Step 3:** Extracellular matrix (ECM) scaffold is washed twice with deionized water and incubated with 80% acetone for 90 minutes to remove residual SDS. After centrifugation at 12,000 rpm for 15 minutes at 4°C, the supernatants are discarded and the ECM scaffold pellets are washed twice with PBS.

**SUPPLEMENTARY FIGURES**


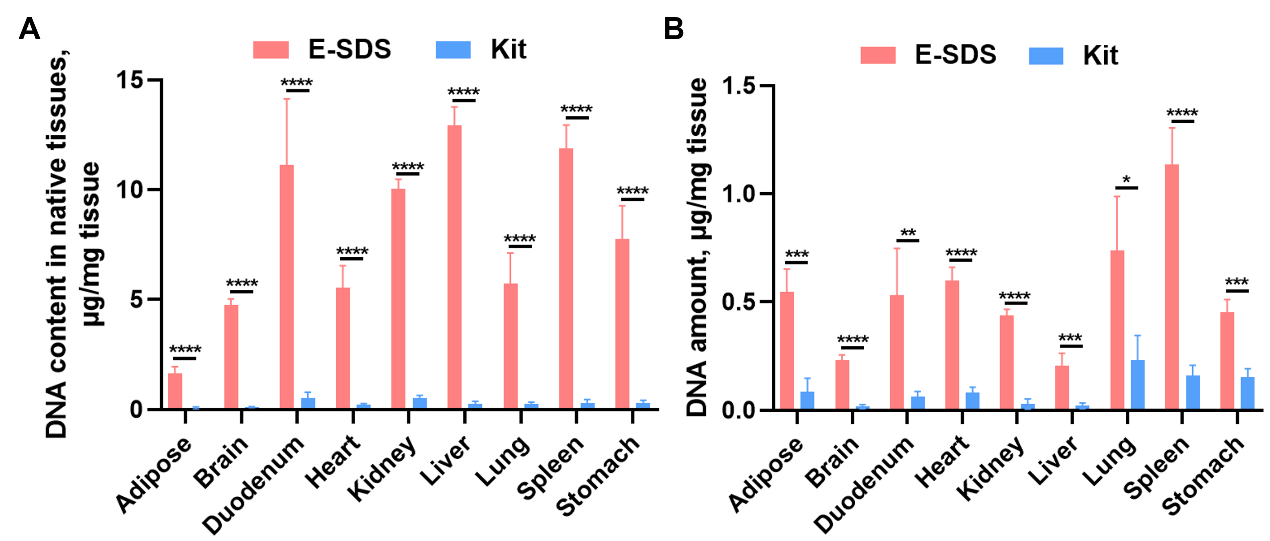


**Figure S1. DND content quantification in native and dECM scaffolds based on E-SDS or commercial kit extraction.** (**A**) Comparison of DNA contents in native tissues using commercial tissue genomic DNA extraction kit and 1^st^ round decellularization solution of E-SDS method. (**B**) Comparison of DNA contents in dECM scaffolds using commercial tissue genomic DNA extraction kit and 2^st^ round decellularization solution of E-SDS method. The absolute DNA content was calculated by multiplying DNA concentration and volume and then dividing by tissue weight. Significance of difference was determined using Student’s *t*-test. **p* < 0.05, ***p* < 0.01, ****p* < 0.001, *****p* < 0.0001, n=4 for each group.


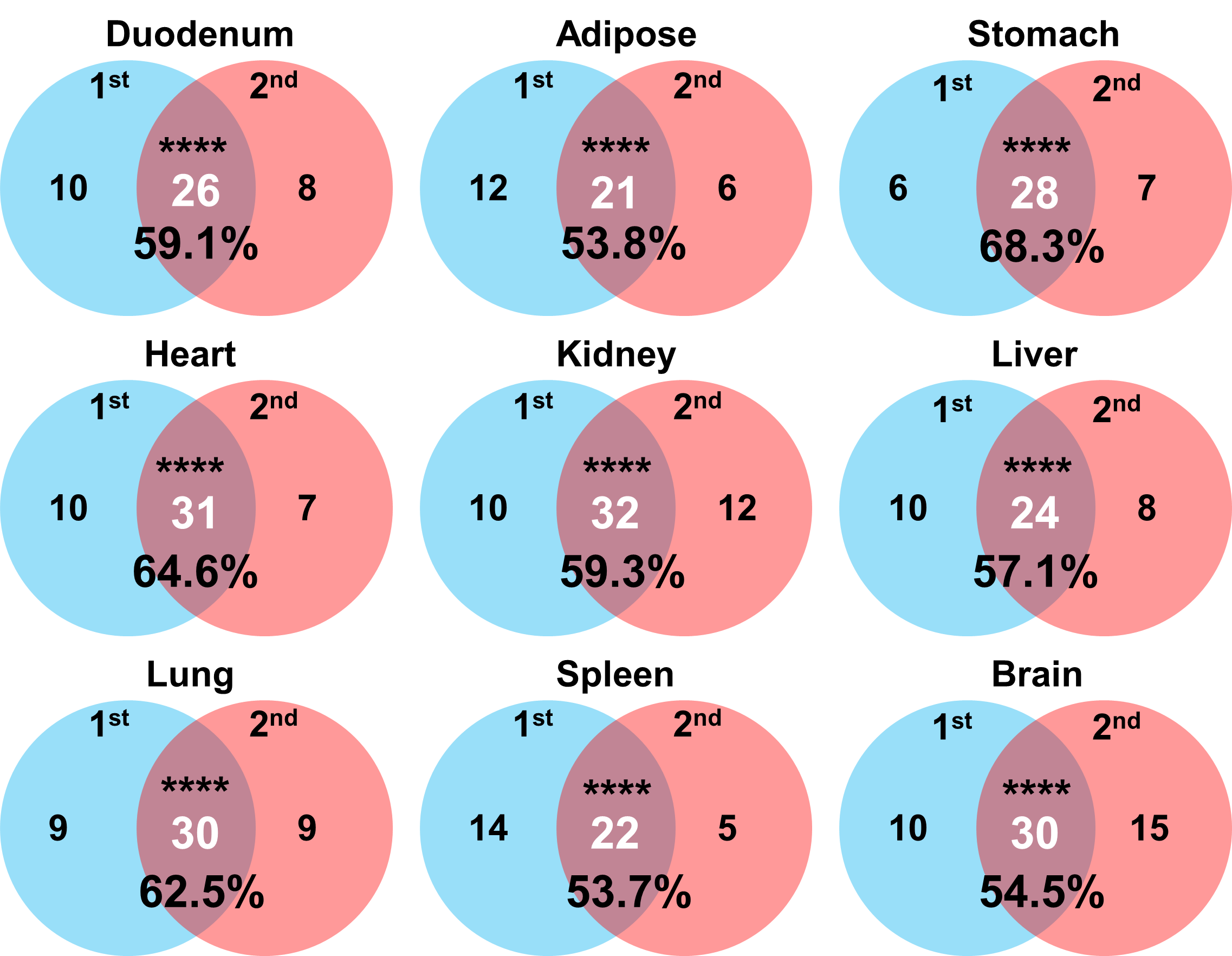


**Figure S2. Venn diagram of the commonly identified matrisome proteins in liver dECM scaffolds obtained by E-SDS pipeline between two replicates.** Percentage was calculated using the number of common matrisome proteins divided by the total number of the identified matrisome proteins in two replicates. Statistical significance was determined using Fisher’s exact test. *****p* < 0.0001.
